# Diazido­bis­(5,5′-dimethyl-2,2′-bipyridyl-κ^2^ *N*,*N*′)cobalt(II) monohydrate

**DOI:** 10.1107/S1600536811011305

**Published:** 2011-03-31

**Authors:** Jaturong Phatchimkun, Narongsak Chaichit

**Affiliations:** a Department of Computer Science, Faculty of Engineering, Vongchavalitkul University, Nakhon Ratchasima 30000, Thailand; b Department of Physics, Faculty of Science and Technology, Thammasat University, Rangsit, Pathumthani 12121, Thailand

## Abstract

In the title compound, [Co(C_12_H_12_N_2_)_2_(N_3_)_2_]·H_2_O, the Co(II) ion is situated on a crystallographic twofold axis and adopts a distorted octa­hedral geometry with the two dmbpy (dmbpy = 5,5′-dimethyl-2,2′-bipyrid­yl) and the two azido ligands in a *cis* arrangement. The solvent water mol­ecule and one methyl group of the dmbpy ligand are disordered over two sets of sites in a 1:1 ratio. The crystal structure is stabilized by intra­molecular C—H⋯N(dmbpy) and inter­molecular O—H⋯N(azide) hydrogen bonds.

## Related literature

For related structures with dmbpy ligands, see: Phatchimkun *et al.* (2009 ▶); van Albada *et al.* (2004 ▶, 2005 ▶); Catalan *et al.* (1995 ▶); Kooijman *et al.* (2002 ▶). For azido complexes, see: Ribas *et al.* (1999 ▶) and references therein. For Co—N bond lengths in azido-containing mononuclear Co(II) complexes, see: Cheng & Hu (2003 ▶). For a description of the Cambridge Structural Database, see: Allen (2002 ▶).
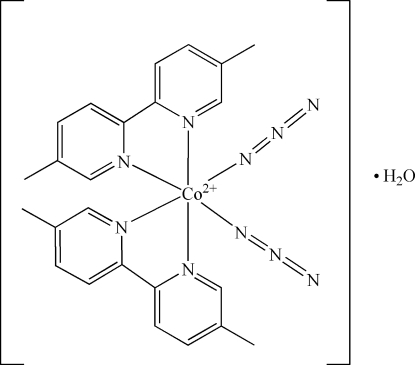


## Experimental

### 

#### Crystal data

[Co(C_12_H_12_N_2_)_2_(N_3_)_2_]·H_2_O
*M* *_r_* = 529.46
Orthorhombic, 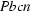

*a* = 17.1030 (3) Å
*b* = 8.5544 (2) Å
*c* = 16.7062 (5) Å
*V* = 2444.22 (10) Å^3^
*Z* = 4
Mo *K*α radiation
μ = 0.74 mm^−1^
*T* = 298 K
0.30 × 0.25 × 0.06 mm

#### Data collection

Nonius KappaCCD diffractometer
Absorption correction: multi-scan (*SADABS*; Sheldrick, 1996 ▶) *T*_min_ = 0.801, *T*_max_ = 0.957
13768 measured reflections
2606 independent reflections
2176 reflections with *I* > 2σ(*I*)
*R*_int_ = 0.022

#### Refinement

*R*[*F* ^2^ > 2σ(*F* ^2^)] = 0.024
*wR*(*F* ^2^) = 0.067
*S* = 1.03
2606 reflections
208 parameters
H atoms treated by a mixture of independent and constrained refinement
Δρ_max_ = 0.16 e Å^−3^
Δρ_min_ = −0.25 e Å^−3^

### 

Data collection: *COLLECT* (Nonius, 2002 ▶); cell refinement: *COLLECT* and *DENZO*/*SCALEPACK* (Otwinowski & Minor, 1997 ▶); data reduction: *DENZO*/*SCALEPACK*; program(s) used to solve structure: *SHELXS97* (Sheldrick, 2008 ▶); program(s) used to refine structure: *SHELXL97* (Sheldrick, 2008 ▶); molecular graphics: *SHELXTL* (Sheldrick, 2008 ▶); software used to prepare material for publication: *SHELXTL*.

## Supplementary Material

Crystal structure: contains datablocks I, global. DOI: 10.1107/S1600536811011305/fj2407sup1.cif

Structure factors: contains datablocks I. DOI: 10.1107/S1600536811011305/fj2407Isup2.hkl

Additional supplementary materials:  crystallographic information; 3D view; checkCIF report

## Figures and Tables

**Table 1:** Selected bond lengths (Å)

Co1—N1	2.0907 (11)
Co1—N2	2.0929 (10)
Co1—N3	2.1095 (11)

**Table 2:** Hydrogen-bond geometry (Å, °)

*D*—H⋯*A*	*D*—H	H⋯*A*	*D*⋯*A*	*D*—H⋯*A*
O1—H1*O*⋯N3^i^	1.05 (5)	1.99 (5)	2.926 (3)	141 (4)
C3—H3⋯O1^ii^	1.00 (2)	2.51 (2)	3.392 (4)	147.1 (14)
C1—H1⋯N3^iii^	0.991 (15)	2.485 (15)	3.1241 (18)	121.9 (11)

Symmetry codes: (i) 
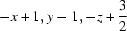
; (ii) 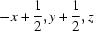; (iii) 
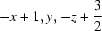
.

**Fig. 1.**
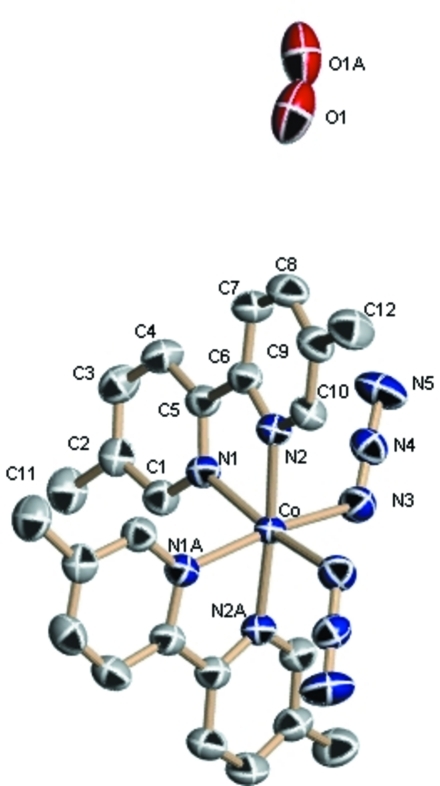
A view of the title structure with the atom-numbering scheme and displacement ellipsoids drawn at the 30% probability level. H atoms have been omitted for clarity.

## supplementary crystallographic information

### Comment

Over the last decades, much attention has been paid on azido bridging complexes in the molecule-based magnet research field because azide anion is not only a good bridging ligand for metal ions (such as Cu(II), Ni(II), Mn(II), Co(II) *etc*) but also an efficient magnetic coupler (Ribas, *et al.*, 1999). On the other hand, azide can act as a monomeric ligand. More than 100 structures of compounds containing the azide anion and cobalt(II) have been reported in the Cambridge Structural Database (CSD; Version 5.29, November 2008 update; Allen, 2002). However, X-ray structures of monomeric compounds with Co^II^ and azide are very rare. Only 16 crystal structures of cobalt(II) azido monomeric complexes have been reported (CSD code: BAWSIC, FURHEF, GURLEK, HIWDOH, HOVVIX, KAVSIJ, LEXXIW, MIRYAO, MONMAE, OHITTEE, PUBXEQ, RAKFUE, RARHAU, RAZHOP, RETDIE, and RUPTIG). Currently, there is no one report of crystal structure containing Co^II^ and 5,5'-dimethyl-2,2'-bipyridyl. Here we report on another monomeric compound, namely [Co(dmbpy)_2_(N_3_)_2_]. H_2_O, where dmbpy is 5,5'-dimethyl-2,2'-bipyridyl.

It is found that Co ion is coordinated by four nitrogen atoms from dmbpy and two azido nitrogen atoms, taking a distorted octahedral geometry. The two bidentate ligands have a *cis* disposition around the metal ion, forming practically perpendicular planes [N1–Co–N1^i^ 89.81 (6), N2–Co–N2^i^ 175.94 (6)°]. The rigidity of these ligands causes the bond angles N1–Co–N2, 78.12 (4) to deviate significantly from orthogonality. This causes the geometry about the Co^II^ ion to deviate slightly from that of an ideal octahedron. The Co–N(dmbpy) bond distances in a complex [2.0916 (12) and 2.1091 (13) Å] are almost the same as those found in the Co(II) compound of [Co^II^(phen)_2_(N_3_)_2_] (2.067 (2)–2.114 (2) Å) (Cheng & Hu, 2003). Good agreement is observed between the Co–*N*(azido) bond distance of 2.1091 (13) Å and those reported (Cheng & Hu, 2003) for azido containing mononuclear cobalt(II) complexes. The crystal structure is stabilized by intramolecular C—H···N and intermolecular O—H···N hydrogen bonds (Table 1).

### Experimental

Preparation of [Co(dmbpy)_2_(N_3_)_2_]. H_2_O. A warm solution of dmbpy (0.181 g, 1.0 mmol) in methanol (15 cm^3^) was added to a hot aqueous solution (10 cm^3^) of Co(CH_3_COO)_2_ (0.123 g, 0.5 mmol). An aqueous solution (10 cm^3^) of NaN_3_ (0.081 g, 1.0 mmol) was then added to the reaction mixture. The pink solution was slowly evaporated at room temperature. Slightly red crystals of [Co(dmbpy)(N_3_)_2_] were deposited. The crystals were filtered off, washed with mother liquor and air-dried. Yield *ca* 75%. (Anal. Calc. for C_24_H_26_CoN_10_O (%): C, 54.44; H, 4.95; N, 26.45. Found: C, 54.08; H, 4.72; N, 26.27). IR (in cm^-1^): n_as_(N_3_) 2009 s, n(C—N) 1605 m, n(C—C) 1584 m.

### Refinement

The water O atom is disordered which site occupancies of 0.5 and 0.5. A l l non-H atom were refined anisotropically. H atoms in aromatic were placed in idealized positions and constrained to ride on their parent atoms, with C–H distances of 0.969–1.02 Å [*U*_iso_ (H)=1.2Ueq (C)]. H atoms in disorder methyl group C(11) were placed at calculated positions, riding on their carrier atoms.

### Crystal data

**Table:**

[Co(C_12_H_12_N_2_)_2_(N_3_)_2_]·H_2_O	*F*(000) = 1096
*M**_r_* = 529.46	*D*_x_ = 1.439 Mg m^−^^3^
Orthorhombic, *P**b**c**n*	Mo *K*α radiation, λ = 0.71073 Å
Hall symbol: -P 2n 2ab	Cell parameters from 7908 reflections
*a* = 17.1030 (3) Å	θ = 0.5–0.6°
*b* = 8.5544 (2) Å	µ = 0.74 mm^−^^1^
*c* = 16.7062 (5) Å	*T* = 298 K
*V* = 2444.22 (10) Å^3^	Plate, red
*Z* = 4	0.30 × 0.25 × 0.06 mm

### Data collection

**Table:**

Nonius KappaCCD diffractometer	2606 independent reflections
Radiation source: fine-focus sealed tube	2176 reflections with *I* > 2σ(*I*)
graphite	*R*_int_ = 0.022
ω scans	θ_max_ = 26.7°, θ_min_ = 2.4°
Absorption correction: multi-scan (*SADABS*; Sheldrick, 1996)	*h* = −21→20
*T*_min_ = 0.801, *T*_max_ = 0.957	*k* = −9→10
13768 measured reflections	*l* = −18→21

### Refinement

**Table:**

Refinement on *F*^2^	Primary atom site location: structure-invariant direct methods
Least-squares matrix: full	Secondary atom site location: difference Fourier map
*R*[*F*^2^ > 2σ(*F*^2^)] = 0.024	Hydrogen site location: inferred from neighbouring sites
*wR*(*F*^2^) = 0.067	H atoms treated by a mixture of independent and constrained refinement
*S* = 1.03	*w* = 1/[σ^2^(*F*_o_^2^) + (0.0369*P*)^2^ + 0.4953*P*] where *P* = (*F*_o_^2^ + 2*F*_c_^2^)/3
2606 reflections	(Δ/σ)_max_ = 0.001
208 parameters	Δρ_max_ = 0.16 e Å^−^^3^
0 restraints	Δρ_min_ = −0.24 e Å^−^^3^

### Special details

**Table:**

Geometry. All e.s.d.'s (except the e.s.d. in the dihedral angle between two l.s. planes) are estimated using the full covariance matrix. The cell e.s.d.'s are taken into account individually in the estimation of e.s.d.'s in distances, angles and torsion angles; correlations between e.s.d.'s in cell parameters are only used when they are defined by crystal symmetry. An approximate (isotropic) treatment of cell e.s.d.'s is used for estimating e.s.d.'s involving l.s. planes.
Refinement. Refinement of *F*^2^ against ALL reflections. The weighted *R*-factor *wR* and goodness of fit *S* are based on *F*^2^, conventional *R*-factors *R* are based on *F*, with *F* set to zero for negative *F*^2^. The threshold expression of *F*^2^ > σ(*F*^2^) is used only for calculating *R*-factors(gt) *etc*. and is not relevant to the choice of reflections for refinement. *R*-factors based on *F*^2^ are statistically about twice as large as those based on *F*, and *R*- factors based on ALL data will be even larger.

### Fractional atomic coordinates and isotropic or equivalent isotropic displacement parameters (Å^2^)

**Table:**

	*x*	*y*	*z*	*U*_iso_*/*U*_eq_	Occ. (<1)
Co1	0.5000	0.74215 (2)	0.7500	0.02599 (9)	
O1	0.4695 (2)	0.2010 (3)	0.7390 (2)	0.0867 (12)	0.50
N1	0.38268 (6)	0.73352 (12)	0.71511 (7)	0.0331 (2)	
N2	0.45546 (6)	0.56878 (12)	0.82573 (6)	0.0324 (2)	
N3	0.47390 (7)	0.91391 (14)	0.83657 (7)	0.0418 (3)	
N4	0.41325 (7)	0.92089 (14)	0.87142 (7)	0.0386 (3)	
N5	0.35504 (8)	0.9289 (2)	0.90686 (9)	0.0648 (4)	
C1	0.35034 (8)	0.81583 (17)	0.65500 (8)	0.0376 (3)	
C2	0.27051 (8)	0.81862 (18)	0.63952 (9)	0.0417 (3)	
C3	0.22267 (9)	0.73661 (18)	0.69218 (10)	0.0473 (4)	
C4	0.25489 (9)	0.65180 (19)	0.75451 (9)	0.0437 (3)	
C5	0.33587 (7)	0.64967 (16)	0.76406 (7)	0.0338 (3)	
C6	0.37688 (7)	0.55243 (15)	0.82431 (7)	0.0331 (3)	
C7	0.33975 (9)	0.44448 (18)	0.87336 (9)	0.0443 (3)	
C8	0.38418 (9)	0.34791 (18)	0.92141 (9)	0.0468 (4)	
C9	0.46490 (9)	0.35865 (16)	0.92140 (8)	0.0394 (3)	
C10	0.49719 (8)	0.47402 (16)	0.87300 (8)	0.0369 (3)	
C11	0.51626 (11)	0.24943 (17)	0.96857 (11)	0.0527 (4)	
C12	0.23851 (11)	0.9052 (2)	0.56797 (11)	0.0560 (4)	
H1	0.3877 (9)	0.8771 (18)	0.6223 (9)	0.045 (4)*	
H3	0.1649 (12)	0.7387 (19)	0.6823 (11)	0.064 (5)*	
H4	0.2211 (9)	0.5925 (19)	0.7907 (9)	0.050 (4)*	
H7	0.2847 (10)	0.434 (2)	0.8706 (10)	0.055 (5)*	
H8	0.3590 (10)	0.2688 (19)	0.9545 (11)	0.057 (5)*	
H10	0.5560 (9)	0.4903 (17)	0.8720 (8)	0.041 (4)*	
H11A	0.4975	0.2431	1.0226	0.079*	0.50
H11B	0.5689	0.2881	0.9685	0.079*	0.50
H11C	0.5150	0.1474	0.9446	0.079*	0.50
H11D	0.4887	0.1705	0.9986	0.079*	0.50
H11E	0.5459	0.3090	1.0125	0.079*	0.50
H11F	0.5566	0.1983	0.9373	0.079*	0.50
H12A	0.2140 (13)	0.826 (3)	0.5282 (14)	0.093 (7)*	
H12B	0.1962 (13)	0.974 (3)	0.5817 (13)	0.087 (7)*	
H12C	0.2796 (13)	0.967 (3)	0.5389 (13)	0.086 (6)*	
H1O	0.514 (3)	0.125 (6)	0.712 (3)	0.106 (16)*	0.50

### Atomic displacement parameters (Å^2^)

**Table:**

	*U*^11^	*U*^22^	*U*^33^	*U*^12^	*U*^13^	*U*^23^
Co1	0.02194 (13)	0.02914 (14)	0.02690 (14)	0.000	0.00172 (8)	0.000
O1	0.135 (4)	0.0515 (13)	0.074 (2)	0.0214 (17)	0.026 (2)	0.0093 (15)
N1	0.0284 (5)	0.0368 (6)	0.0340 (6)	0.0012 (4)	0.0010 (4)	−0.0002 (4)
N2	0.0306 (6)	0.0345 (6)	0.0320 (5)	−0.0013 (4)	0.0014 (4)	−0.0006 (4)
N3	0.0409 (6)	0.0431 (7)	0.0414 (6)	−0.0021 (5)	0.0078 (5)	−0.0071 (5)
N4	0.0370 (6)	0.0434 (6)	0.0354 (6)	0.0076 (5)	−0.0039 (5)	−0.0028 (5)
N5	0.0394 (7)	0.0930 (12)	0.0621 (9)	0.0146 (7)	0.0087 (7)	−0.0108 (8)
C1	0.0353 (7)	0.0410 (7)	0.0364 (7)	0.0038 (6)	0.0008 (6)	0.0008 (6)
C2	0.0362 (7)	0.0451 (8)	0.0440 (8)	0.0079 (6)	−0.0047 (6)	−0.0027 (6)
C3	0.0291 (7)	0.0580 (10)	0.0548 (9)	0.0023 (6)	−0.0041 (6)	−0.0017 (7)
C4	0.0307 (7)	0.0520 (8)	0.0483 (8)	−0.0034 (6)	0.0019 (6)	0.0018 (7)
C5	0.0302 (6)	0.0368 (7)	0.0345 (6)	−0.0006 (5)	0.0017 (5)	−0.0041 (5)
C6	0.0297 (6)	0.0373 (7)	0.0323 (6)	−0.0021 (5)	0.0027 (5)	−0.0033 (5)
C7	0.0352 (7)	0.0524 (9)	0.0453 (8)	−0.0074 (6)	0.0037 (6)	0.0057 (7)
C8	0.0505 (9)	0.0481 (9)	0.0418 (8)	−0.0096 (7)	0.0055 (6)	0.0096 (7)
C9	0.0478 (8)	0.0387 (7)	0.0316 (7)	0.0000 (6)	−0.0007 (6)	0.0006 (6)
C10	0.0353 (7)	0.0390 (7)	0.0365 (7)	0.0003 (6)	−0.0009 (5)	0.0017 (6)
C11	0.0652 (11)	0.0488 (9)	0.0441 (9)	0.0043 (7)	−0.0062 (8)	0.0107 (7)
C12	0.0451 (9)	0.0679 (12)	0.0551 (10)	0.0106 (8)	−0.0099 (8)	0.0100 (9)

### Geometric parameters (Å, °)

**Table:**

O1—O1^i^	1.108 (8)	C5—C4	1.3943 (19)
O1—H1O	1.10 (5)	C2—C3	1.391 (2)
Co1—N1^i^	2.0907 (11)	C2—C12	1.509 (2)
Co1—N1	2.0907 (11)	C8—C9	1.383 (2)
Co1—N2^i^	2.0929 (10)	C8—H8	0.974 (18)
Co1—N2	2.0929 (10)	C10—C9	1.3903 (19)
Co1—N3	2.1095 (11)	C10—H10	1.016 (16)
Co1—N3^i^	2.1095 (12)	C9—C11	1.505 (2)
N2—C10	1.3379 (17)	C4—C3	1.384 (2)
N2—C6	1.3515 (16)	C4—H4	0.978 (16)
N1—C1	1.3454 (17)	C11—H11A	0.9600
N1—C5	1.3505 (17)	C11—H11B	0.9600
N4—N5	1.1604 (17)	C11—H11C	0.9600
N4—N3	1.1909 (16)	C11—H11D	0.9650
C1—C2	1.3898 (19)	C11—H11E	1.0271
C1—H1	0.990 (15)	C11—H11F	0.9703
C6—C7	1.3884 (19)	C12—H12B	0.96 (2)
C6—C5	1.4822 (18)	C12—H12C	1.01 (2)
C7—C8	1.380 (2)	C12—H12A	1.04 (2)
C7—H7	0.946 (17)	C3—H3	1.00 (2)
			
O1^i^—O1—H1O	58 (3)	C9—C8—H8	119.1 (10)
N1^i^—Co1—N1	175.95 (6)	N2—C10—C9	124.16 (13)
N1^i^—Co1—N2^i^	78.13 (4)	N2—C10—H10	115.8 (8)
N1—Co1—N2^i^	98.96 (4)	C9—C10—H10	120.0 (8)
N1^i^—Co1—N2	98.96 (4)	C8—C9—C10	116.33 (13)
N1—Co1—N2	78.13 (4)	C8—C9—C11	122.76 (14)
N2^i^—Co1—N2	89.76 (6)	C10—C9—C11	120.88 (14)
N1^i^—Co1—N3	92.09 (5)	C3—C4—C5	119.25 (14)
N1—Co1—N3	90.72 (5)	C3—C4—H4	120.1 (9)
N2^i^—Co1—N3	170.08 (4)	C5—C4—H4	120.6 (9)
N2—Co1—N3	90.12 (4)	C9—C11—H11A	109.5
N1^i^—Co1—N3^i^	90.72 (5)	C9—C11—H11B	109.5
N1—Co1—N3^i^	92.09 (5)	H11A—C11—H11B	109.5
N2^i^—Co1—N3^i^	90.12 (4)	C9—C11—H11C	109.5
N2—Co1—N3^i^	170.08 (4)	H11A—C11—H11C	109.5
N3—Co1—N3^i^	91.70 (7)	H11B—C11—H11C	109.5
C10—N2—C6	118.56 (11)	C9—C11—H11D	114.9
C10—N2—Co1	126.30 (9)	H11A—C11—H11D	46.2
C6—N2—Co1	115.13 (8)	H11B—C11—H11D	134.5
C1—N1—C5	119.08 (11)	H11C—C11—H11D	64.5
C1—N1—Co1	125.75 (9)	C9—C11—H11E	110.7
C5—N1—Co1	114.76 (9)	H11A—C11—H11E	61.3
N5—N4—N3	178.48 (15)	H11B—C11—H11E	50.7
N1—C1—C2	123.49 (13)	H11C—C11—H11E	139.4
N1—C1—H1	115.0 (9)	H11D—C11—H11E	102.5
C2—C1—H1	121.5 (9)	C9—C11—H11F	114.4
N2—C6—C7	120.89 (12)	H11A—C11—H11F	136.0
N2—C6—C5	115.11 (11)	H11B—C11—H11F	59.1
C7—C6—C5	123.89 (12)	H11C—C11—H11F	51.8
C8—C7—C6	119.32 (13)	H11D—C11—H11F	108.3
C8—C7—H7	121.3 (10)	H11E—C11—H11F	104.9
C6—C7—H7	119.2 (10)	C2—C12—H12B	112.6 (13)
N4—N3—Co1	123.67 (10)	C2—C12—H12C	112.9 (13)
N1—C5—C4	120.84 (12)	H12B—C12—H12C	108.6 (18)
N1—C5—C6	115.39 (11)	C2—C12—H12A	109.6 (13)
C4—C5—C6	123.71 (12)	H12B—C12—H12A	104.3 (18)
C1—C2—C3	116.83 (13)	H12C—C12—H12A	108.5 (17)
C1—C2—C12	120.81 (14)	C4—C3—C2	120.40 (14)
C3—C2—C12	122.36 (14)	C4—C3—H3	121.7 (10)
C7—C8—C9	120.64 (13)	C2—C3—H3	117.8 (10)
C7—C8—H8	120.2 (11)		
			
N1^i^—Co1—N2—C10	−7.07 (11)	N1—Co1—N3—N4	32.12 (12)
N1—Co1—N2—C10	170.07 (11)	N2—Co1—N3—N4	−46.01 (12)
N2^i^—Co1—N2—C10	70.87 (10)	N3^i^—Co1—N3—N4	124.24 (13)
N3—Co1—N2—C10	−99.21 (11)	C1—N1—C5—C4	−2.10 (19)
N1^i^—Co1—N2—C6	174.06 (9)	Co1—N1—C5—C4	170.99 (11)
N1—Co1—N2—C6	−8.79 (9)	C1—N1—C5—C6	175.16 (11)
N2^i^—Co1—N2—C6	−108.00 (9)	Co1—N1—C5—C6	−11.75 (14)
N3—Co1—N2—C6	81.92 (9)	N2—C6—C5—N1	4.27 (16)
N2^i^—Co1—N1—C1	−88.45 (11)	C7—C6—C5—N1	−172.02 (13)
N2—Co1—N1—C1	−176.30 (11)	N2—C6—C5—C4	−178.57 (13)
N3—Co1—N1—C1	93.73 (11)	C7—C6—C5—C4	5.1 (2)
N3^i^—Co1—N1—C1	2.00 (11)	N1—C1—C2—C3	3.0 (2)
N2^i^—Co1—N1—C5	98.99 (9)	N1—C1—C2—C12	−176.10 (14)
N2—Co1—N1—C5	11.14 (9)	C6—C7—C8—C9	0.4 (2)
N3—Co1—N1—C5	−78.82 (9)	C6—N2—C10—C9	−0.1 (2)
N3^i^—Co1—N1—C5	−170.56 (9)	Co1—N2—C10—C9	−178.91 (10)
C5—N1—C1—C2	−0.7 (2)	C7—C8—C9—C10	2.1 (2)
Co1—N1—C1—C2	−172.92 (10)	C7—C8—C9—C11	−175.95 (15)
C10—N2—C6—C7	2.80 (19)	N2—C10—C9—C8	−2.3 (2)
Co1—N2—C6—C7	−178.24 (10)	N2—C10—C9—C11	175.74 (13)
C10—N2—C6—C5	−173.61 (11)	N1—C5—C4—C3	2.4 (2)
Co1—N2—C6—C5	5.35 (14)	C6—C5—C4—C3	−174.66 (13)
N2—C6—C7—C8	−3.0 (2)	C5—C4—C3—C2	0.1 (2)
C5—C6—C7—C8	173.09 (13)	C1—C2—C3—C4	−2.7 (2)
N1^i^—Co1—N3—N4	−144.98 (12)	C12—C2—C3—C4	176.44 (16)

Symmetry codes: (i) −*x*+1, *y*, −*z*+3/2.

### Hydrogen-bond geometry (Å, °)

**Table:**

*D*—H···*A*	*D*—H	H···*A*	*D*···*A*	*D*—H···*A*
O1—H1O···N3^ii^	1.05 (5)	1.99 (5)	2.926 (3)	141 (4)
C3—H3···O1^iii^	1.00 (2)	2.51 (2)	3.392 (4)	147.1 (14)
C1—H1···N3^i^	0.991 (15)	2.485 (15)	3.1241 (18)	121.9 (11)

Symmetry codes: (ii) −*x*+1, *y*−1, −*z*+3/2; (iii) −*x*+1/2, *y*+1/2, *z*; (i) −*x*+1, *y*, −*z*+3/2.
